# The role of weight- and appearance-related discrimination on eating disorder symptoms among adolescents and emerging adults

**DOI:** 10.1186/s12889-021-11756-y

**Published:** 2021-09-26

**Authors:** Caroline Cohrdes, Claudia Santos-Hövener, Katja Kajikhina, Heike Hölling

**Affiliations:** Department of Epidemiology and Health Monitoring, Robert Koch Institute, Nordufer 20, 13353 Berlin, Germany

**Keywords:** Eating disorder symptoms, Adolescents, Emerging adults, Sex differences, Discrimination, Social media use, Body image, Self-efficacy, Social support, KIGGS study

## Abstract

**Background:**

Eating disorder symptoms (EDs) have been discussed as a prominent problem among late adolescent girls with serious health risks and long-term consequences. However, there is a lack of population-based evidence on EDs comprising the age range from early adolescence to emerging adulthood as well as considering both females and males equally. Additionally, the differential role of a comprehensive set of several relevant risk factors and particularly weight- and appearance-related discrimination warrants further attention. Thus, we aimed to contribute to a better understanding of sex- and age-related differences in associations between discrimination experience and other relevant personal risk factors (body image, social media use, self-efficacy, social support) with EDs. Furthermore, we were interested in the exploration of underlying mechanisms enhancing the risk of EDs by taking discrimination experience into account.

**Methods:**

Based on a logistic regression model, we investigated associations between weight- and appearance-related discrimination and EDs while controlling for other relevant personal risk factors in a subsample of *N* = 8504 adolescents and emerging adults (54.4% female, mean age = 20.71 years, *SD* = 4.32 years) drawn from a German representative health survey (KiGGS Wave 2). In a second step, we investigated the mediating role of discrimination experience between the other risk factors and EDs with the help of a path model.

**Results:**

While controlling for other relevant personal risk factors, weight- and appearance-related discrimination was significantly related to EDs. Whereas the risk of EDs was significantly enhanced in males and emerging adults frequently experiencing weight-related discrimination, adolescents showed a higher risk of EDs when experiencing appearance-related discrimination. Moreover, discrimination experience partly explained the associations between body image dissatisfaction, low self-efficacy, high media use and ED symptoms.

**Conclusions:**

The results highlight weight- and appearance-related discrimination as one central factor to be considered in the pathogeneses of EDs and underpin the need for discrimination prevention as well as the promotion of adaptive coping with discrimination experience to reduce the risk of developing ED symptoms. Males and emerging adults need particular attention when facing weight-related discrimination whereas risk constellations and EDs particularly affecting females need further investigation.

## Background

Individuals showing symptoms of disordered eating (e.g., regularly skipping meals or fasting, binge eating and/or purging) are at risk of developing severe mental and physical problems, including increased mortality and suicide [1, 2]. While the worldwide incidence rate of eating disorders (EDs) has remained relatively stable at approximately 1 to 4% over the past 20 years, there has been an increase in subthreshold symptomatology (i.e., the presence of symptoms without reaching clinical significance) in general and among late-adolescent girls in particular [3, 4]). The results from a recent nationally representative survey of children and adolescents living in Germany substantiate these findings: a total of 19.8% of 11- to 17-year-old children and adolescents showed ED symptoms, with a significantly higher prevalence in females compared to males and a higher prevalence in adolescents aged 14 to 17 years compared to those aged 11 to 13 years [5]. Explanatory approaches highlight the role of social media in the development and maintenance of body image norms and corresponding eating disorder symptoms by endorsing a thin ideal in late adolescent and emerging adult females [6, 7]. To date, the question of how far relations between the frequent use of social media and ED symptoms are indeed a phenomenon, particularly in adolescent girls, is unclear and needs to be investigated in more detail.

### The role of age and sex for developing eating disorder symptoms

In general, evidence suggests that EDs most commonly develop during adolescence with an age at onset between 13 and 18 years but often occur even earlier for anorexia nervosa and later for bulimia nervosa and binge eating [1, 8]. One study indicates that the age of 14 is a critical phase for adolescent girls by showing that risk factors for EDs (e.g., body dissatisfaction) are most distinctly present at that time [9]. The results on the probability of developing EDs in transition to emerging adulthood are mixed and await further evidence. So far, the majority of studies yield evidence of increases in ED incidence as well as subthreshold ED symptoms with older age [8, 10]. Moreover, if already present during adolescence, ED symptoms have a relatively high risk of persisting or worsening in transition to emerging adulthood [8, 11].

Familial (e.g., climate, emotional responsiveness), social (e.g., support, discrimination) and individual factors (e.g., self-efficacy, body dissatisfaction) have been identified as risk factors for the incidence as well as the persistence of ED symptoms [5, 12, 13]. However, only a few studies have addressed age- and sex-specific risk factors for ED symptoms as yet, and studies including males are generally underrepresented [14, 15]. The few studies that examined both sexes suggest that body dissatisfaction is more strongly related to female ED symptoms, while low levels of self-esteem and social support are more strongly associated with ED symptoms in boys [16, 17]. Moreover, in epidemiological studies, the relevance of the social environment in shaping psychosocial risk factors for ED symptoms, such as the idealization of a certain body image, has been neglected so far [18, 19]. In particular, the role of weight- and appearance-related discrimination could contribute to the understanding of ED pathogenesis and warrants more public and scientific attention. Within this context, associations of ED symptoms with social media activity need to be considered, since it represents an increasingly popular medium to express or compare oneself to others as well as to receive positive or negative (i.e., discriminatory) feedback.

### Associations between discrimination experiences and eating disorder symptoms

Perceived discrimination initiates a heightened physiological and psychological stress response and thereby enhances negative health outcomes as well as unhealthy behaviours [20]. Within the field of body image research, the term discrimination refers to negative feedback in the forms of insults, cruel or contemptuous remarks, also often labelled “teasing” [21–23]. The results from a meta-analysis suggest that weight- and appearance-related discrimination in particular play a significant role in body dissatisfaction and disordered eating with moderate effect sizes [21]. Moreover, discrimination experiences occur in diverse social contexts, such as with family members, peers or strangers, but may be of greater importance with close others [24, 25]. Children [26], adolescents [27] and young adults [19, 28] reporting experiences of weight- or appearance-related discrimination are at equivalent higher risk of ED symptoms as well as other psychological comorbidities. Frequent maladaptive social comparison has been discussed as one major factor affecting associations between negative feedback and ED symptoms [28–30]: individuals receiving negative feedback more frequently tend to compare their own body image to presumably more attractive or idealized others (upward comparison), leading to dissatisfaction with one’s own appearance.

### Body image, self-efficacy, social media use and perceived social support as risk factors for eating disorder symptoms

Evidence is accumulating that media exposure in terms of volume and frequency predicts body dissatisfaction, thin body ideals, and ED symptoms among adolescent and emerging adult females [31, 32]. In addition, findings suggest that social media use in particular can come with both benefits (e.g., increased levels of self-esteem, perceived social support) and costs (e.g., increased exposure to teasing or bullying, social isolation [29]). However, the level of activity or passivity as well as the content of social media use has been suggested to help understand the direction and strength of such effects [29]. As opposed to other media, the unique characteristics of contemporary social media technologies are real-time interactivity and advanced self-disclosure options via various modalities (e.g., chat, video, animation [30]). Therefore, opportunities for social comparison and feedback become omnipresent and require guidance in healthy and self-protective handling [33].

In addition, predisposing characteristics such as a dissatisfied body image [34], low self-efficacy (i.e., one’s belief in one’s own capacities to reach a goal, master challenges [35]) or low social support [36] need to be taken into account when investigating discrimination associations with EDs, especially within a social media context. For example, evidence suggests that adolescents with body image dissatisfaction may be highly susceptible to weight- or appearance-related media content and show larger effects on psychological well-being compared to those with body image satisfaction [34]. Discrimination experience regarding weight or appearance has also been related to lower levels of self-efficacy [35]. High self-efficacy, on the contrary, has been related to better psychological well-being in general [37] as well as to less eating disorders, in particular [5, 27]. Furthermore, social support has been found to have significant effects on the onset, trajectory, and recovery from EDs [17, 25, 38], as well as on the consequences from discrimination experiences [39]. The importance and pressure of being thin among key interpersonal or medial social supports such as family or peers predicted the onset of ED symptoms, whereas positive perceptions of support served as a powerful resource for prevention or recovery [25]. In conclusion, although further longitudinal research is needed, the findings suggest that self-efficacy and social support offer the potential to affect the handling of discrimination experiences and thereby protect children and adolescents against later ED and other psychopathological symptoms [37].

### Hypotheses

The present study is addressing a research gap between male and female EDs in a non-clinical sample of adolescents and young adults and aims to increase knowledge about the role of weight- and appearance-related discrimination experiences as well as the interplay with other relevant personal characteristics (e.g., self-efficay, body image). We thereby aim to add new insights into the understanding of associations between ED symptoms and risk factors as well as into potentially helpful fields of prevention.

Based on the research summarized, we hypothesized that discrimination experiences because of weight or appearance predict adolescent and emerging adult ED symptoms while controlling for body image, social media use, self-efficacy and social support (Hypothesis 1; H1). We moreover expected age and sex differences in the associations between discrimination experiences and other relevant personal factors (body image, social media use, self-efficacy, social support) and ED symptoms. More precisely, we expected females and emerging adults to be at higher risk of eating disorder symptoms in case of discrimination experience than males and adolescents (H2a). Moreover, we expected to replicate indications of males to be at higher risk for ED symptoms in case of low self-efficacy or low social support and of girls to be at higher risk in case of body dissatisfaction and high social media use (H2b). Due to missing knowledge on age-specific risks, we explored age-related differences in associations with ED symptoms (Exploratory Question 1; EQ1). In addition, we explored the extent to which discrimination experiences based on weight and appearance further explain the associations between body image, social media use, self-efficacy and perceived social support with ED symptoms (EQ2).

## Methods

### Sample and procedure

The present research is based on data from the German Interview and Examination Survey for Children and Adolescents (KiGGS), a regularly conducted national health monitoring survey combining a cross-sectional (KiGGS Baseline: 2003–2006; Wave 1: 2011–2013, Wave 2: 2014–2017) with a longitudinal part (KiGGS Cohort Study) at the German National Health Institute. At KiGGS Wave 2, representative samples were randomly drawn from 167 sample points reflecting Germany’s regional structure based on municipal population registries (more details can be found in [40, 41]). We applied several measures (e.g., easily understandable information for diverse population groups, questionnaire translations in various languages, home visits to encourage participation, free phone support, incentives such as shopping vouchers) to ensure that the sample reflects the composition of the target population at the best [40, 41]. In total, 15,023 cross-sectional participants from a few months to 17 years of age and 10,853 longitudinal participants from 10 to 31 years of age (61.5% of the KiGGS baseline sample) took part in the interview of KiGGS Wave 2 and answered paper-pencil questionnaires. All participants aged 11 or older answered the questionnaires individually, and parents answered a parental questionnaire including information about their socioeconomic status.

The present cross-sectional analyses include the total of *N =* 8504 participants that fell into the age range of interest (adolescents and young adults from 14 to 31 years; mean age = 20.71*, SD =* 4.32; 54.4% female) and have answered the questions related to the present study’s interest. Thereof, *n* = 2426 participants were adolescents ranging in age between 14 and 17 years (mean age = 15.52, *SD* = 1.09, 51.5% female), and *n =* 6078 were emerging adults ranging in age between 18 and 31 years (mean age = 22.78*, SD =* 3.25, 55.2% female). Table 1 gives a detailed overview of the present sample characteristics and variables under study.

**Table 1:** Sample Characteristics of *N* = 8504 (Mean Age = 20.71, *SD* = 4.32; 54.4% Female) Adolescents and Emerging Adults of KiGGS-2 in Total, Grouped by Sex (Male, Female) and Age (Underage, Full Age)

	Total	Sex		Age	
		Male	Female	14–17 years	18+ years
		***n*** = 3870	***n*** = 4634	*n* = 2426	*n* = 6078
	% [95% *CI*]	% [95% *CI*]	% [95% *CI*]	% [95% *CI*]	% [95% *CI*]
ED symptoms	18.52 [17.71–19.35]	10.96 [10.02–11.97]	24.94 [23.71–26.20]	21.04 [19.46–22.73]	17.63 [16.09–18.61]
**Weight-related discrimination**					
Never	72.85 [71.90–73.79]	75.89 [74.52–77.21]	70.33 [68.99–71.63]	74.33 [72.28–76.81]	72.44 [71.30–73.55]
Seldom	14.26 [13.54–15.02]	13.93 [12.87–15.06]	14.54 [13.56–15.59]	15.09 [13.53–16.81]	13.91 [13.07–14.81]
Sometimes	8.71 [8.13–9.33]	7.57 [6.78–8.44]	9.67 [8.85–10.55]	7.57 [6.45–8.87]	9.08 [8.38–9.83]
Often	3.08 [2.73–3.47]	1.90 [1.59–2.48]	3.99 [3.46–4.54]	1.58 [1.10–2.26]	3.53 [3.10–4.03]
Very often	1.08 [0.88–1.33]	0.62 [0.42–0.92]	1.47 [1.16–1.86]	1.41 [0.97–2.07]	1.02 [0.79–1.31]
**Appearance-related discrimination**					
Never	69.16 [68.17–70.13]	72.13 [70.69–73.52]	66.67 [65.30–68.02]	67.83 [65.65–69.93]	69.61 [68.44–70.75]
Seldom	18.05 [17.25–18.89]	17.45 [16.28–18.67]	18.56 [17.47–19.71]	20.21 [18.43–22.11]	17.49 [16.56–18.47]
Sometimes	9.20 [8.60–9.83]	8.13 [7.31–9.03]	10.08 [9.25–10.99]	8.47 [7.27–9.83]	9.37 [8.66–10.13]
Often	2.57 [2.26–2.93]	1.68 [1.31–2.13]	3.32 [1.84–3.88]	2.24 [1.65–3.03]	2.59 [2.22–3.02]
Very often	1.02 [0.83–1.26]	0.62 [0.42–0.92]	1.36 [1.06–1.74]	1.26 [0.84–1.88]	0.94 [0.72–1.21]
**Parental SES**					
Low	10.75 [10.11–11.44]	10.11 [9.19–11.11]	11.29 [10.41–12.25]	10.48 [9.33–11.78]	10.87 [10.10–11.66]
Moderate	61.08 [60.00–62.11]	60.62 [59.06–62.22]	61.46 [60.04–62.86]	60.66 [58.69–62.59]	61.25 [60.00–62.52]
High	28.16 [27.21–29.13]	29.27 [27.84–30.73]	27.24 [25.97–28.55]	28.86 [27.08–30.70]	27.88 [26.76–29.03]
**Body image**					
Just right	38.44 [37.42–39.48]	40.42 [38.90–41.96]	36.77 [35.39–38.16]	44.31 [42.37–46.27]	36.06 [34.87–37.27]
A little too thin	12.49 [11.82–13.21]	19.97 [18.75–21.24]	6.16 [5.50–6.89]	14.15 [12.84–15.58]	11.82 [11.04–12.66]
Much too thin	1.79 [1.53–2.09]	2.73 [2.27–3.29]	1.00 [0.74–1.32]	1.85 [1.39–2.46]	1.76 [1.46–2.13]
A little too thick	40.59 [39.56–41.63]	32.30 [30.85–33.77]	47.63 [46.19–49.06]	34.90 [33.05–36.80]	42.90 [41.67–44.15]
Much too thick	6.68 [6.17–7.23]	4.58 [3.97–5.27]	8.46 [7.69–9.29]	4.78 [4.01–5.70]	7.45 [6.82–8.13]
**Self-efficacy**^**1**^	65.72 [65.41–66.01]	68.19 [67.76–68.62]	63.65 [63.24–64.07]	65.17 [64.58–65.75]	65.93 [65.57–66.29]
**Social media use**^**2**^	1.59 [1.55–1.62]	1.40 [1.35–1.44]	1.78 [1.73–1.83]	1.91 [1.83–1.99]	1.49 [1.45–1.57]
**Social support**^**3**^	91 [78–100]	88 [72–100]	94 [81–100]	91 [78–100]	94 [78–100]

*Note.*^1^Self-efficacy is indicated by its mean and 95% confidence interval, min = 0 and max = 100. ^2^Social support is indicated by its median and interquartile range, min = 0 and max = 100. ^3^Social media use is indicated by the average time spent in hours a day and 95% confidence interval, min = 0 (never) and max = 5 (five hours or more)

### Measures

#### Eating disorder (ED) symptoms

We used the German version of the SCOFF screening tool for detecting symptomatic expressions of EDs [42]. The questionnaire includes 5 questions assessing the core symptoms of anorexia and bulimia nervosa (“Do you make yourself **S**ick because you feel uncomfortably full? Do you worry that you have lost **C**ontrol over how much you eat? Have you recently lost more than **O**ne stone (14 lb/6k g) in a 3-month period? Do you believe yourself to be **F**at when others say you are too thin? Would you say that **F**ood dominates your life?”) answered on a dichotomous scale (yes vs. no). Responding positively to at least two out of the five questions indicates ED symptomatology [42]. Correspondingly, answers were dichotomized to indicate ED symptomatology (1) or no ED symptomatology (0).

#### Weight- and appearance-related discrimination

Among other discrimination experiences, participants were asked whether and how often they experienced discrimination due to their weight or appearance (“It may happen that you are discriminated (treated unfairly or being disadvantaged). How often have you had this experience due to your weight/appearance?“) on an ordinal scale from 0 (never) to 4 (very often). The questions are adapted from the German Youth Institute Foreigners Survey [43].

#### Parental socioeconomic status (SES)

Since socioeconomic status (SES) has been discussed as potentially affecting the occurrence of EDs [44] but is not a focus of the present study, we also took parental SES as a control variable into account. Parental SES was indicated by an index score comprising the education and occupational status and the net equalized income of both parents (see [44] for a detailed description). In the present analyses, the SES was categorized based on the German population distribution containing three categories that reflect the ranking of children or adolescents by the social status of the households in which they live: low (lower quintile), medium (2nd to 4th quintile), and high (upper quintile).

#### Body image (BI)

Body self-image was assessed using the following inhouse-developed question and corresponding five answer options: “Do you think that you are just the right weight/a bit too thin/a bit too thick/much too thin/much too thick”. For regression and path analyses, answers were categorized with 0 (just right), 1 (slightly too thin or thick) and 2 (much too thin/thick) to indicate deviations from a preferred body image in two severity levels.

#### Self-efficacy (SE)

Self-efficacy was measured with the German 10-item short form of the general self-efficacy scale [45]. Responses are given on a 4-point rating scale from 1 (not at all true) to 4 (exactly) indicating the belief that one can perform a novel or difficult task or cope with adversity (e.g., “I am confident that I could deal efficiently with unexpected events”). Answers were summarized to a sum score and transformed to a standardized scale with a minimum of 0 and a maximum of 100, in line with then original coding scheme [45]. The internal consistency was good with α = .86.

#### Social media use (SMU)

As an indication of the average amount of social media use, participants answered the question “How much time do you spend in social networks (e.g., Facebook) on average per day?” on a 6-point rating scale with the response options 0 (never), 1 (one hour), 2 (two hours), 3 (three hours), 4 (four hours), 5 (five hours or more).

#### Social support (SUP)

Perceived levels of social support were assessed with a German version of the social support survey [46] as used in [47]. The scale comprises 8 items (e.g., “Someone you can count on to listen to you when you need to talk”, “Someone who hugs you”) answered on a 5-point rating scale from 0 (none of the time) to 4 (all of the time). Analogous to the original coding scheme, answers were summarized to a sum score and transformed to a standardized scale with a minimum of 0 and a maximum of 100 [46]. The internal consistency was very good with α = .90.

### Statistical analyses

Analyses were performed with STATA version 15.1 [48]. To answer Hypotheses 1, 2 and our exploratory question EQ1, we conducted two logistic regression models. In Model 1, we predicted ED symptoms by weight- and appearance-related discrimination experiences and while controlling for age, sex, parental SES and other relevant personal factors that have already been identified as relevant covariates in previous research (BI, SE, SMU, SUP). To analyse systematic age and sex differences in the association between discrimination experiences as well as other relevant personal factors and ED symptoms (H2), we next included the interaction terms of age as well as sex with weight- and appearance-related discrimination, BI, SE, SMU and SUP in Model 2. Ninety-five percent confidence intervals represent Clopper-Pearson intervals based on the assumption of binomial distribution. Thereafter, we compared the goodness of fit between Model 1 and Model 2 by means of a Likelihood-Ratio (LR) test. Furthermore, we calculated contrasts for significant differences in the simple slopes of associations between discrimination experience and ED symptoms, separated by age and sex. To answer our exploratory question EQ2, we conducted a path analysis based on the structural equation model technique and maximum-likelihood estimation to investigate the mediating role of discrimination experiences in associations between relevant personal factors (BI, SE, SMU, SUP) and ED symptoms (outcome) while controlling for age, sex and parental SES as relevant covariates of EDs. For significant indirect relations, we calculated the amount of variance of the total effect explained by the indirect effect [49]. Missing values varied between 0 and 1.1% (parental SES) and were excluded list-wise from regression and path analyses.

## Results

In line with H1, the results from logistic regression suggest that the odds of ED symptoms are 1.57 to 2.05 respectively 1.26 to 2.15 times higher in adolescents and emerging adults with weight- and appearance-related discrimination experiences than in those without such experiences (Table 2, Model 1). This finding remains significant even after controlling for other relevant personal factors (BI, SE, SMU, SUP), as suggested in H1.

**Table 2:** Results from Logistic Regression Analyses Predicting Adolescent and Emerging Adult Eating Disorder Symptoms (*N* = 8117) by Discrimination Experiences (Model 1), while Controlling for Age, Sex, Parental SES, Body Image, Self-efficacy, Social Media Use, Social Support

	Eating disorder symptoms (EDs)		
Predictors Model 1	***OR***	95% ***CI***	***p***
Age	**0.97**	**0.96–0.98**	**<.001**
Sex			
Male vs. Female	**2.48**	**2.16–2.84**	**<.001**
Parental SES			
low vs. medium	1.08	0.88–1.31	.445
low vs. high	1.20	0.97–1.49	.098
Body image			
Just right vs. little too thin/thick	**2.88**	**2.46–3.37**	**<.001**
Just right vs. much too thin/thick	**5.56**	**4.41–7.00**	**<.001**
Self-efficacy	**0.99**	**0.98–0.99**	**<.001**
Social media use	**1.15**	**1.09–1.20**	**<.001**
Social support	0.99	0.99–1.00	.139
Weight-related discrimination			
Never vs. seldom	**1.57**	**1.32–1.85**	**<.001**
Never vs. sometimes	**2.05**	**1.67–2.52**	**<.001**
Never vs. often	**1.81**	**1.30–2.51**	**<.001**
Never vs. very often	**1.81**	**1.02–3.23**	**.044**
Appearance-related discrimination			
Never vs. seldom	**1.26**	**1.07–1.48**	**.005**
Never vs. sometimes	**1.34**	**1.08–1.64**	**.007**
Never vs. often	0.95	0.65–1.37	.776
Never vs. very often	**2.15**	**1.19–3.89**	**.011**

*Note.* OR = adjusted odds ratio, SES = socioeconomic status. Significant results at *p* < .05 are highlighted in boldface. Self-efficacy and social support are indicated by a sum score ranging between 0 (min) and 100 (max). Social media use is indicated by the average time spent in hours a day, ranging from 0 (never) to 5 (five hours or more). Model 1 Pseudo *R*^2^ = .14

**Fig. 1:**
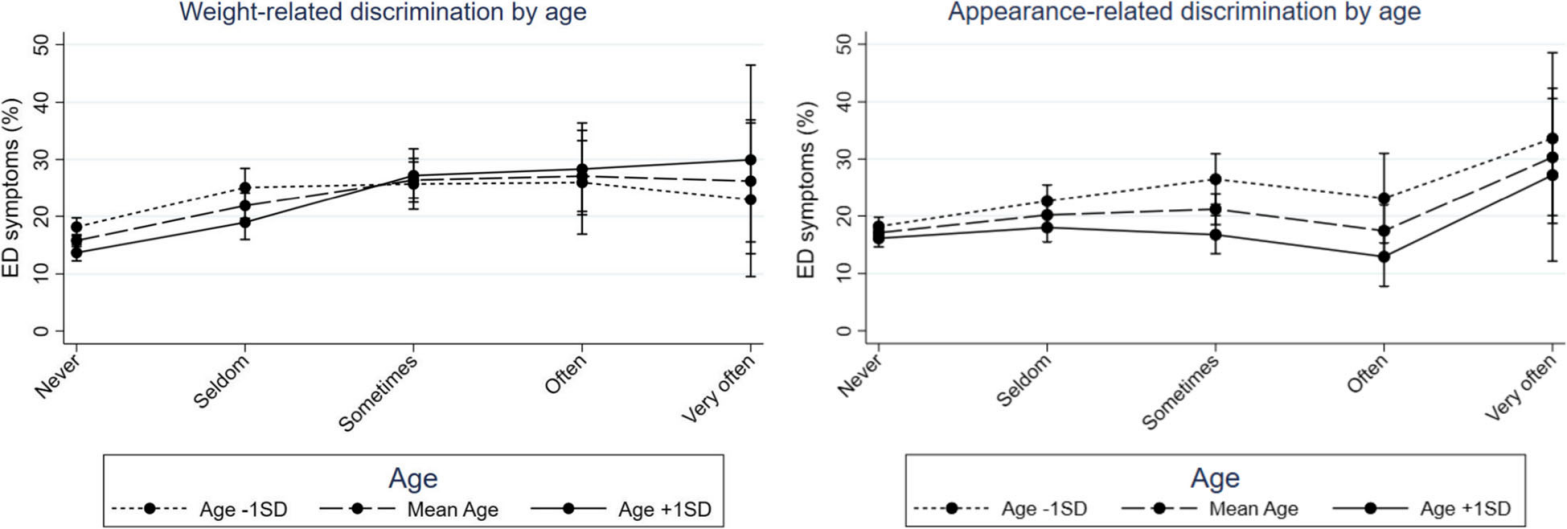
Simple slopes of weight- and appearance-related discrimination predicting eating disorder (ED) symptoms at 1 *SD* below the mean age (16.3), the mean age (20.7) and 1 *SD* above the mean age (25.1). Error bars represent 95% *CI*s

**Fig. 2:**
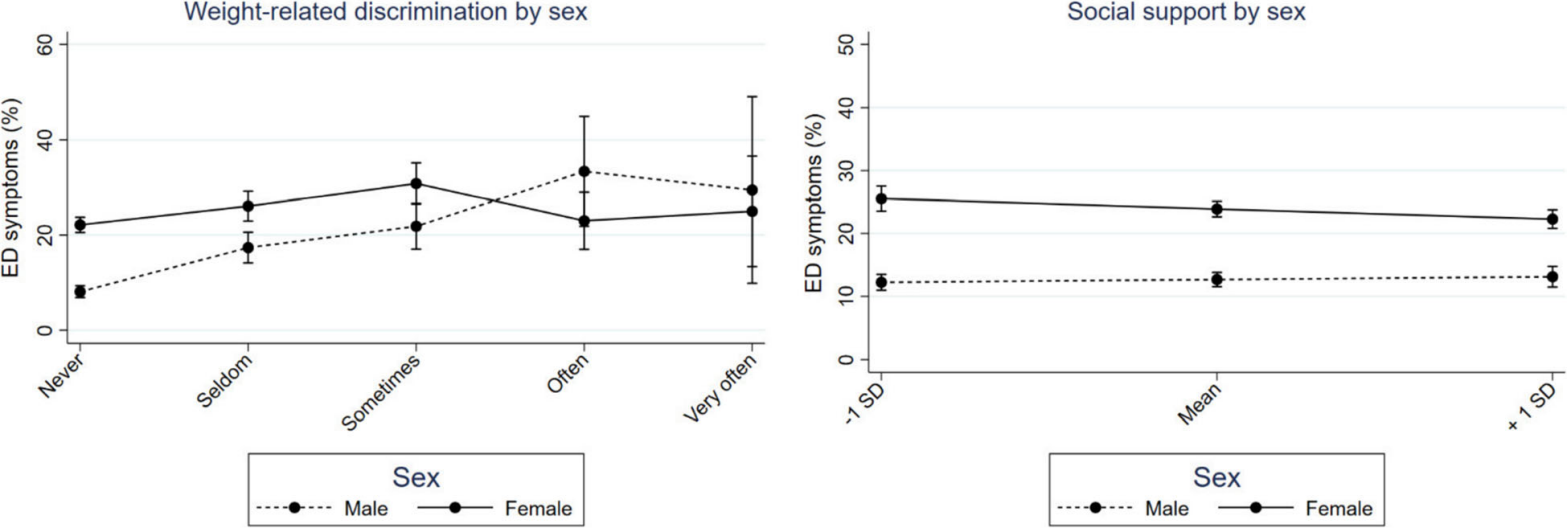
Simple slopes of weight-related discrimination and social support for male and female participants predicting eating disorder (ED) symptoms at 1 *SD* below the mean (86.2), at the mean (72.6) and 1 *SD* above the mean (100.0). Error bars represent 95% *CI*s

The results moreover indicate systematic age and sex differences in the associations between weight- and appearance-related discrimination and ED symptoms, as suggested by H2a and H2b (Table 3, Model 2). While associations between weight-related discrimination and ED symptoms were stronger the older participants were, the opposite was the case for appearance-related discrimination (Fig. 1, Table 4). Young adults reporting weight-related discrimination often to very often were on average 15.5% more likely to show EDs than those without such experiences (Table 4). However, differences between participants one *SD* below the mean age, at the mean age and one *SD* above the mean age were significant when experiencing weight- or appearance-related discrimination sometimes, only (Table 3, Model 2). Associations between discrimination experiences and EDs at the other discrimination levels showed high variation, as indicated by the confidence intervals in Fig. 1 and Table 3. Furthermore, the more frequently males experienced weight-related discrimination the stronger were the associations with ED symptoms (Fig. 2, Table 3). The found sex differences were significant at each of the discrimination levels but associations reversed at the sometimes level: while associations of weight-related discrimination with EDs increased at first and remained relatively stable thereafter for females, associations steadily increased for males (Fig. 2). Males experiencing often or very often weight-related discrimination were on average about 22% more likely to show EDs than those without such experiences (Table 4).

**Table 3:** Results from Logistic Regression Analyses Predicting Adolescent and Emerging Adult Eating Disorder Symptoms (*N* = 8117) by Discrimination Experiences Complemented by Age and Sex Interactions with Discrimination Experiences (Model 2), while Controlling for Age, Sex, Parental SES, Body Image, Self-efficacy, Social Media Use, Social Support

	Eating disorder symptoms (EDs)		
Predictors Model 2	***OR***	95% ***CI***	***p***
Age	0.98	0.89–1.09	.806
Sex			
Male vs. Female	**8.62**	**3.33–22.33**	**<.001**
Parental SES			
low vs. medium	1.07	0.89–1.30	.492
low vs. high	1.18	0.95–1.47	.125
Body image			
Just right vs. little too thin/thick	**3.64**	**1.65–8.05**	**.001**
Just right vs. much too thin/thick	**3.73**	**1.15–11.99**	**.027**
Self-efficacy	**0.99**	**0.98–1.00**	**<.001**
Social media use	**1.38**	**1.09–1.75**	**.008**
Social support	1.00	0.98–1.02	.804
Weight-related discrimination			
Never vs. seldom	**2.67**	**1.13–6.30**	**.025**
Never vs. sometimes	1.20	0.41–3.50	.743
Never vs. often	2.04	0.33–12.80	.443
Never vs. very often	0.89	0.05–17.55	.939
Appearance-related discrimination			
Never vs. seldom	1.80	0.79–4.09	.158
Never vs. sometimes	**4.53**	**1.53–13.46**	**.007**
Never vs. often	4.66	0.63–34.19	.131
Never vs. very often	3.20	0.14–74.93	.468
***Age interactions***			
Age × BI right vs. little too thin/thick	0.98	0.94–1.01	.1201
Age × BI right vs. much too thin/thick	1.01	0.95–1.06	.789
Age × Self-efficacy	1.00	0.99–1.00	.598
Age × Social media use	0.99	0.98–1.00	.171
Age × Social support	1.00	0.99–1.00	.952
Age × seldom weight-related discrimination	1.00	0.96–1.04	.872
Age × sometimes weight-related discrimination	**1.06**	**1.00–1.11**	**.046**
Age × often weight-related discrimination	1.06	0.97–1.14	.181
Age × very often seldom weight-related discrimination	1.09	0.95–1.24	.223
Age × seldom appearance-related discrimination	0.98	0.95–1.02	.337
Age × sometimes appearance-related discrimination	**0.94**	**0.89–0.99**	**.026**
Age × often appearance-related discrimination	0.93	0.85–1.01	.103
Age × very often appearance-related discrimination	0.98	0.84–1.13	.771
***Sex interactions***			
Sex × BI right vs. little too thin/thick	1.41	0.99–2.01	.054
Sex × BI right vs. much too thin/thick	1.32	0.81–2.15	.272
Sex × Self-efficacy	1.00	0.98–1.01	.518
Sex × Social media use	0.96	0.86–1.06	.389
Sex × Social support	**0.99**	**0.98–0.99**	**.019**
Sex × seldom weight-related discrimination	**0.51**	**0.35–0.73**	**<.001**
Sex × sometimes weight-related discrimination	**0.48**	**0.31–0.75**	**.001**
Sex × often weight-related discrimination	**0.17**	**0.08–0.33**	**<.001**
Sex × very often weight-related discrimination	**0.23**	**0.07–0.79**	**.020**
Sex × seldom appearance-related discrimination	1.04	0.73–1.47	.827
Sex × sometimes appearance-related discrimination	0.97	0.62–1.53	.900
Sex × often appearance-related discrimination	0.98	0.42–2.34	.977
Sex × very often appearance-related discrimination	1.28	0.35–4.70	.709

*Note.* OR = adjusted odds ratio, SES = socioeconomic status, BI = body image. Significant results at *p* < .05 are highlighted in boldface. Self-efficacy and social support are indicated by a sum score ranging between 0 (min) and 100 (max). Social media use is indicated by the average time spent in hours a day, ranging from 0 (never) to 5 (five hours or more). Model 2 Pseudo *R*^2^ = .15

**Table 4:** Contrasts and Significance of Differences between the Simple Slopes for Discrimination Experiences as well as for Social Support on Eating Disorder Symptoms, Grouped by Sex and Age and as Shown in Figs. 1 and 2

	Sex		Age		
	Male	Female	Age − 1 ***SD***	Mean age	Age + 1 ***SD***
**Comparisons**	**Contrast (*SE*)**	**Contrast (*SE*)**	**Contrast (*SE*)**	**Contrast (*SE*)**	**Contrast (*SE*)**
***Weight-related discrimination on EDs***					
Never vs. seldom	.09** (.017)	.04* (.019)	.07** (.019)	.06** (.013)	.05** (.017)
Never vs. sometimes	.13** (.024)	.09** (.025)	.08** (.025)	.11** (.017)	.14** (.026)
Never vs. often	.24** (.060)	.01 (.034)	.08 (.048)	.11** (.032)	.15** (.043)
Never vs. very often	.20* (.097)	.03 (.062)	.05 (.069)	.10 (.055)	.16* (.085)
***Appearance-related discrimination on EDs***					
Never vs. seldom	–		.05** (.016)	03** (.011)	.02 (.015)
Never vs. sometimes	–		.08** (.025)	.04** (.015)	.01 (.019)
Never vs. often	–		.05 (.042)	.01 (.024)	−.03 (.028)
Never vs. very often	–		.15* (.077)	.13* (.053)	.11 (.078)
***Social support on EDs***					
- 1 SD vs. Mean	−.01 (.014)	−.05* (.023)	–		
- 1 SD vs. + 1 SD	.01 (.012)	−.07** (.019)	–		

*Note*. EDs = Eating disorder symptoms. Age − 1 *SD* = 16.3 years, mean age = 20.7 years, age + 1 *SD* = 35.1 years. Significant results are indicated by **p < .01 and *p < .05

Additionally, sex and age interactions with the other personal factors were significant for social support only (H2b). Levels of perceived social support were significantly related to ED symptoms for females but unrelated for males (Fig. 2, Table 4). Females with low social support were on average about 6% more likely to show EDs compared to their counterparts (Table 4). There were no significant interactions between the participant’s age and BI, SE, SMU or SUP on ED symptoms (EQ1). A significant LR test supported the assumption that Model 2 fits the data significantly better than Model 1, χ^2^ = 73.66, *p* < .001.

**Fig. 3:**
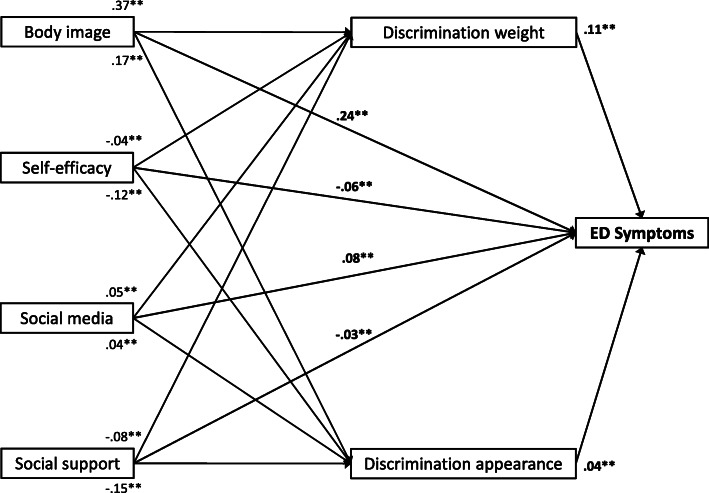
Standardized regression coefficients indicating the *total effects* of discrimination experiences and other relevant personal factors on eating disorder (ED) symptoms as a result of a weighted path model. Participants’ age, sex and parental SES were included as control variables, although they are not presented in the figure. The figure shows significant total paths at **p < .01 and *p < .05 only. Model fit indices: *R*^2^ = .27, χ^2^ = 6151.19, *CFI* = 1.00, *RMSEA* < .001

The results from path analysis indicated excellent fit of the proposed model to the present data, χ^2^ = 6151.19, *p* < .001, *CFI* = 1.00, *RMSEA* < .001 [50]. As shown in Fig. 3, the present findings replicate the results from logistic regression, suggesting direct relations between BI, SE, SMU, and SUP with discrimination experience as well as ED symptoms. While high levels of SI and SUP were associated with less discrimination and ED symptoms, a too thin or thick BI as well as high levels of SMU were associated with more frequent discrimination as well as ED symptoms (Fig. 3, Table 5). In particular, BI showed a comparatively strong relation with weight-related and SE as well as SUP with appearance-related discrimination.

**Table 5:** Direct, Indirect and Total Standardized Effects as Results from Path Model on Adolescent and Young Adult Eating Disorder Symptoms (*N* = 8117) Predicted by Age, Sex, Parental SES, Body Image, Self-efficacy, Social Media Use, Social Support and Mediated via Discrimination Experiences

	Direct effect		Indirect effect		Total effect	
Predictors	β (***SE***)	***p***	β (***SE***)	***p***	β (***SE***)	***p***
***Outcome: ED symptoms (R***^***2***^ ***= .13)***						
Age	**−.05 (.001)**	**<.001**	<.01 (<.001)	.352	**−.04 (.001)**	**<.001**
Sex	**.14 (.009)**	**<.001**	**.01 (.001)**	**<.001**	**.16 (.009)**	**<.001**
Parental SES	.02 (.007)	.067	−.01 (.001)	**<.001**	.01 (.007)	.190
Body image	**.19 (.007)**	**<.001**	**.05 (.003)**	**<.001**	**.24 (.007)**	**<.001**
Self-efficacy	**−.05 (.001)**	**<.001**	**−.01 (<.001)**	**<.001**	**−.06 (.001)**	**<.001**
Social media use	**.07 (.003)**	**<.001**	**.01 (.001)**	**.018**	**.08(.003)**	**<.001**
Social support	−.02 (.001)	.093	**−.02 (.001)**	**<.001**	**−.03 (.001)**	**.002**
Weight-related discrimination	**.11 (.006)**	**<.001**	–		**.11 (.006)**	**<.001**
Appearance-related discrimination	**.04 (.006)**	**.001**	–		**.04 (.006)**	**.001**
***Outcome: Weight-related discrimination (R***^***2***^ ***= .17)***						
Age	.01 (.002)	.229	–		.01 (.002)	.229
Sex	**.07 (.018)**	**<.001**	–		**.07 (.018)**	**<.001**
Parental SES	**−.03 (.015)**	**.002**	–		**−.03 (.015)**	**.002**
Body image	**.37 (.014)**	**<.001**	–		**.37 (.014)**	**<.001**
Self-efficacy	**−.04 (.001)**	**<.001**	–		**−.04 (.001)**	**<.001**
Social media use	**.05 (.007)**	**<.001**	–		**.05 (.007)**	**<.001**
Social support	**−.08 (.001)**	**<.001**	–		**−.08 (.001)**	**<.001**
***Outcome: Appearance-related discrimination (R***^***2***^ ***= .10)***						
Age	−.01 (.002)	.928	–		−.01 (.002)	.928
Sex	**.07 (.018)**	**<.001**	–		**.07 (.018)**	**<.001**
Parental SES	**−.04 (.014)**	**<.001**	–		**−.04 (.014)**	**<.001**
Body image	**.17 (.011)**	**<.001**	–		**.17 (.011)**	**<.001**
Self-efficacy	**−.12 (.001)**	**<.001**	–		**−.12 (.001)**	**<.001**
Social media use	**.04 (.006)**	**<.001**	–		**.04 (.006)**	**<.001**
Social support	**−.15 (.001)**	**<.001**	–		**−.15 (.001)**	**<.001**

*Note*. ED = eating disorder; SES = socioeconomic status. Significant results at *p* < .05 are highlighted in boldface. Model fit indices: *R*^2^ = .27, χ^2^ = 6151.19, *CFI* = 1.00, *RMSEA* < .001

**Fig. 4:**
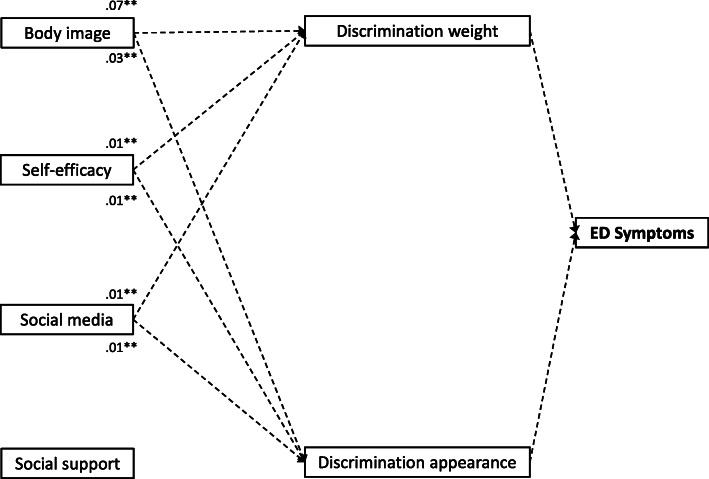
Standardized regression coefficients indicating the indirect effects of personal factors (age, sex, body image, parental SES, self-efficacy, social media use and perceived social support) on eating disorder (ED) symptoms mediated via discrimination experiences as a result of a weighted path model. Participants’ age, sex and parental SES were included as control variables, although they are not presented in the figure. The figure shows significant indirect paths at **p < .01 and *p < .05 only. Model fit indices: *R*^2^ = .27, χ^2^ = 6151.19, *CFI* = 1.00, *RMSEA* < .001

In addition, the results suggest indirect relations between BI, SE and SMU with ED symptoms mediated via both weight- and appearance-related discrimination as addressed in EQ2 (Fig. 4, Table 5). The variances explained of the total effect by the indirect effect were 16.9% for BI, 7.3% for SE and 8.0% for SMU regarding weight-related discrimination and 2.8% for BI, 8.0% for SE and 2.0% for SMU regarding appearance-related discrimination, indicating that experiencing discrimination explains a relatively high proportion of variance in the association between BI and ED symptoms in particular.

## Discussion

With the present study, we aimed to investigate the frequency and impact of weight- and appearance-related discrimination on ED symptoms in an adolescent and emerging adult German population sample and to contribute to a better understanding of sex- and age-related differences in associations between discrimination experience and other relevant personal factors with ED symptoms. Furthermore, we were interested in the exploration of underlying mechanisms enhancing the risk of ED symptoms, especially for those with discrimination experience.

In support of previous findings [2, 19, 21] and confirming H1, weight and appearance-related discrimination was associated with a higher risk of ED symptoms in adolescents and emerging adults. This finding remained robust even after controlling for other relevant personal factors. By taking interactions with the sex and age of participants into account, the present findings additionally show that frequent weight-related discrimination is associated with ED symptoms, particularly in males. Thus, we can conclude that although ED symptoms are generally more prevalent in females, the risk is increased in males experiencing weight-related discrimination often to very often. One possible explanation is the relatively high burden and shame due to stigmatization issues and feelings of compromised masculinity, as already discussed within the context of help-seeking-barriers [14]. Similar to depressive symptoms, classical male role models may bar the way to disclose problems or symptoms related to eating that may be overlooked and need to be addressed by, for instance, mental health literacy media campaigns [51]. Furthermore, we found that weight-related discrimination is not only more frequently reported by emerging adults but also more strongly related to ED symptoms. Associations between appearance-related discrimination and ED symptoms, on the contrary, were stronger for adolescents. These findings relativize general assumptions of females in transition to emerging adulthood being at higher risk of both ED symptoms and discrimination experience, as addressed in H2a [4, 5, 52].

In addition, the results are contradictory to previous indications of sex-differential risk factors for ED symptoms (H2b [6, 7];) and suggest that body image, self-efficacy and social media use do not differ significantly in their associations with ED symptoms between males and females or between adolescents and emerging adults. One explanation for the mixed findings is based on a sex-invariant operationalization of body image in various studies. Boys and girls might be similarly dissatisfied with their body but in different ways: whereas males have been more concerned with increasing specific muscular (upper) body parts, females have been more concerned with decreasing the overall size of their body [17, 53]. The present findings reflect this difference indirectly by showing that a higher proportion of females feel a little or much too thick, while males more frequently feel a little or much too thin.

One exception is represented by the finding that low social support was associated with enhanced ED symptoms in females, but not as expected in males (H2b [17]). Explanations of why social support differed in the association with ED symptoms between females and males require further research. Findings can be interpreted in line with previous indications of sex differences in the perceived level or type of social support (e.g., emotional [54]), the relative importance of specific social support (e.g., family [36]) and varying effect sizes regarding the impact on diverse socially directed psychopathological symptoms (e.g., externalizing problems [39]). For example, females might perceive and expect a higher level of social support and thus also rely more on these resources in critical situations so that the absence of (expected) social support is perceived as more harmful compared to males with generally lower levels of (expected) social support. However, these post hoc hypotheses cannot be explored using the present data and require further investigation.

Findings are also contradictory to indications of sex differences in associations between social media use and ED symptoms. Based on previous findings, one would have expected that female adolescents and emerging adults are more attracted to social media than males and that higher social media use is more strongly related to ED symptoms as a consequence of thin body idealization and maladaptive social comparison [29, 30, 32]. Based on the present findings, the time spent with social media alone seems not to be a specific risk factor for females and should be extended by further information such as the content, activity level or motivation for usage. The exploration of age differences in associations between relevant personal factors and ED symptoms (EQ1) revealed no significant results, suggesting that adolescents and emerging adults are similarly affected by perceptions of body image and social support or the level of self-efficacy and media use.

The results moreover extend previous findings on associations between discrimination and ED symptoms by taking discrimination as an explanatory factor into account (EQ2). Indeed, both weight- and appearance-related discrimination explained a significant proportion of variance in associations between a dissatisfied body image, low self-efficacy and high media use with ED symptoms. Thus, there seems to be a relatively frequent co-occurrence of discrimination experience with relevant perceptual (body image, self-efficacy) and behavioural (social media use) risk factors for ED symptoms. The extent to which discrimination leads to adverse self-perceptions or health behaviour such as biased body image or upward social comparison on social media cannot be answered based on the present research and requires longitudinal investigations.

### Strengths and limitations

The present findings provide new insights into the role of discrimination as well as its interplay with other relevant risk factors and their impact on ED symptoms in adolescents and young adults. Based on a large population-based sample of adolescents and emerging adults between the ages of 14 and 31 years old, we performed differentiated analyses according to various groups as well as of combined effects, offering entry points for further investigations and targeted public health measures. However, the present findings should be qualified by the following considerations. First, although the present subsample is drawn from a representative German sample, it does not allow representative statements but rather population-oriented approximations. Next, some of the investigated factors were measured rather globally and lack additional information. For example, we have no information about how (e.g., passive, active) and why (e.g., self-expression, communication) social media is used or whether discrimination was experienced in the context of social media or elsewhere nor its source (e.g., peers, family). This information is necessary to directly link both to each other and should be considered in future studies. Moreover, single item measures of discrimination tended to underestimate the strength of the relationship between discrimination and ED symptoms [21] so that using more comprehensive instruments might add value to the understanding and estimation of the impact on ED symptoms. However, the fact that we found significant direct and mediated effects from discrimination and other personal risk factors on ED symptoms substantiates their significance. Last, age differentiations based on cross-sectional data and await further longitudinal evidence to draw conclusions on the development of ED symptoms and corresponding risk factors in transition to emerging adulthood.

## Conclusions

The present findings add new insights into group-specific effects of discrimination on EDs and thereby offer targeted entry points for prevention. In the case of often to very often weight-related discrimination, the odds of ED symptoms were higher for males and emerging adults, suggesting that these groups need particular attention and care to avoid ED manifestations. Public health measures should promote mental health literacy and self-recognition ability of unhealthy body ideals or behaviours not only tailored to (slimness-oriented) girls and women but also to (muscularity-oriented) boys and men. Apart from that, we found that low levels of perceived social support seem to be particularly detrimental for ED symptoms in adolescent girls. This may be a good starting point for peer-oriented support intervention as well as for studies on the further identification and exploration of mechanisms explaining differences in ED risks and symptoms for females compared to males.

Overall, the results highlight weight- and appearance-related discrimination as one central factor to be considered in the pathogeneses of EDs regardless of the age and sex, and underpin the need for discrimination prevention as well as the promotion of adaptive coping with discrimination experience to reduce the risk of developing ED symptoms. This becomes particularly important considering the finding that discrimination experiences often co-occur with major risk factors of EDs such as body image dissatisfaction or low self-efficacy and thus can function as a warning signal and a hub for prevention approaches at the same time. Important areas to address are the home as well as the social environment by suggestions on how to discourage discriminatory comments, decrease pressure of assimilation to a thin body image, provide support and responsiveness and promote acceptance.

## Data Availability

The datasets generated and/or analysed during the current study are not publicly available because informed consent from study participants did not cover public deposition of data but a scientific use file containing the KiGGS Wave 2 data is available on reasonable request from the Health Monitoring’ Research Data Centre at the Robert Koch Institute in Berlin, Germany (e-mail: fdz@rki.de).

## Abbreviations

ED: eating disorder
KIGGS: German Health Interview and Examination Survey for Children and Adolescents
H: hypothesis
EQ: exploratory question
SE: standard error
CI: confidence interval
SES: socioeconomic status
BI: body image
SE: self-efficacy
SMU: social media use
SUP: social support
